# Burst of reactive oxygen species in pedicel-mediated fruit abscission after carbohydrate supply was cut off in longan (*Dimocarpus longan*)

**DOI:** 10.3389/fpls.2015.00360

**Published:** 2015-05-26

**Authors:** Ziqin Yang, Xiumei Zhong, Yan Fan, Huicong Wang, Jianguo Li, Xuming Huang

**Affiliations:** ^1^Physiological Laboratory for South China Fruits, College of Horticulture, South China Agricultural University, Guangzhou, China; ^2^Tropical Crops Genetic Resources Institute, Chinese Academy of Tropical Agricultural Sciences, Danzhou, China; ^3^Section of Fruit Crops, Dongguan Agricultural Research Center, Dongguan, China

**Keywords:** fruit abscission, carbohydrate stress, reactive oxygen species, plasma membrane-bound NADPH oxidase, cellulase, longan

## Abstract

Cutting off carbohydrate supply to longan (*Dimocarpus longan* Lour.) fruit by girdling and defoliation or by detachment induced 100% abscission within a few days. We used these treatments to study the involvement of reactive oxygen species (ROS) in fruit abscission. Girdling plus defoliation decreased sugar concentrations in the fruit and pedicel and depleted starch grains in the chloroplasts in the cells of abscission zone. Prior to the occurrence of intensive fruit abscission, there was a burst in ROS in the pedicel, which peaked at 1 day after treatment (DAT), when H_2_O_2_ in the abscission zone was found to be chiefly located along the plasma membrane (PM). H_2_O_2_ was found exclusively in the cell walls 2 DAT, almost disappeared 3 DAT, and reappeared in the mitochondria and cell walls 4 DAT. Signs of cell death such as cytoplasm breakdown were apparent from 3 DAT. The burst of ROS coincided with a sharp increase in the activity of PM-bound NADPH oxidase in the pedicel. At the same time, activities of antioxidant enzymes including superoxide dismutase (SOD), catalase, and peroxidase (POD) were all increased by the treatment and maintained higher than those in the control. Accompanying the reduction in H_2_O_2_ abundance, there was a sharp decrease in PM-bound NADPH oxidase activity after 1 DAT in the treated fruit. H_2_O_2_ scavenger dimethylthiourea (DMTU, 1 g L^–1^) significantly inhibited fruit abscission in detached fruit clusters and suppressed the increase in cellulase activity in the abscission zone. These results suggest that fruit abscission induced by carbohydrate stress is mediated by ROS. Roles of ROS in regulating fruit abscission were discussed in relation to its subcellular distribution.

## Introduction

Carbohydrates serve as the “hard currency” in plants, representing the costs for various biological functions including growth, maintenance, and defense. Fruit are net importers of carbohydrates from the tree reserves or leaf photosynthesis (Mehouachi et al., 2000; Hieke et al., 2002; Iglesias et al., 2003). Fruit trees generally produce more fruitlets than they can support to harvest, and fruit abscission is a normal physiological event during fruit development due to a self-regulatory mechanism to reduce fruit load (Bangerth, 2000). However, under adverse conditions, such as shading (Yuan and Huang, 1988; Zhou et al., 2008; Li et al., 2013), high temperatures (Atkinson et al., 2001; Gazit and Degani, 2002), or abrupt temperature fluctuations (Yang et al., 2010), this mechanism may cause excessive fruit abscission. According to Lakso et al. (2006), environmental factors affect fruit abscission based on the carbohydrate supply–demand balance, and higher carbohydrate availability reduces sensitivities to abscission-inducing stresses or fruit-thinning chemicals. Mehouachi et al. (1995) suggested the existence of a threshold carbohydrate concentration in citrus below which fruit shedding was intensified.

There is limited evidence on how a shortage of carbohydrates initiates the activity of the abscission zone leading to fruit shedding. Gómez-Cadenas et al. (2000) found that defoliation increased the concentration of the ethylene precursor 1-aminocyclopropane-1-carboxylic acid (ACC) and abscisic acid (ABA). They suggested that these hormones participate in the self-regulatory mechanism that adjusts fruit load depending on the availability of carbohydrates. Iglesias et al. (2006) observed high ethylene evolution in the fruit and the pedicel and intensive fruit abscission after the fruit stalk was girdled. While the hormone ethylene is a well-known hormone that triggers fruit abscission (Taylor and Whitelaw, 2001; Meir et al., 2010), there is much less information about roles of reactive oxygen species (ROS) in abscission regulation.

Reactive oxygen species is involved in the responses of plants to stresses and is generated by a number of mechanisms including NADPH oxidation catalyzed by plasma membrane (PM)-bound NADPH oxidase (Cheeseman, 2007; Tripathy and Oelmüller, 2012). Reports about the roles ROS in fruit abscission have been inconsistent. Lai et al. (2001) found that H_2_O_2_ reduced wax apple abscission under low temperatures, while Ueda et al. (1991) reported that H_2_O_2_ stimulated abscission of bean petioles under high light levels independent of ethylene. Sakamoto et al. (2008) showed that H_2_O_2_ was involved in salt-induced abscission of pepper petioles and that it acted downstream of ethylene in signaling abscission. Botton et al. (2011) suggested that an increase in sugar concentration in the cortex of apple fruit serves as an initial senescing signal that induced H_2_O_2_ and ethylene production, causing abortion of seed, reduction in IAA export and fruit drop.

Longan (*Dimocarpus longan* Lour.) is a tropical fruit tree that generally sets heavily and requires thinning to produce large fruit, although natural fruit abscission occurs during fruit development. The fruit are borne in multi-fruit panicles. Unlike apple, whose fruit abscission is highly predictable with obvious dominant central fruit and weak side fruit, longan fruit within a panicle are similar in size and vigor and it is difficult to predict which and when fruit will abscise. In this study, longan fruit clusters were starved for carbohydrates by girdling and defoliating, or detaching the fruit clusters. These treatments induced 100% fruit abscission within a few days, providing a convenient experimental system to study signals involved in fruit abscission. Using this system, we examined the occurrence and roles of ROS in regulating abscission under carbohydrate stress.

## Materials and Methods

### Materials and Treatments

The study was carried out during the mid stage of fruit development (50–60 days after anthesis), after the early wave of fruit drop had ended and before the rapid aril (flesh) growth initiated. The on-tree experiments involved girdling and defoliation treatments, which were performed on 12- to 14-year-old “Chuliang” trees at the South China Agricultural University or Dongguan Agricultural Research Center. The off-tree experiments used detached fruit clusters harvested from these trees.

Effectiveness of girdling plus defoliation in inducing fruit abscission was examined. Twenty bearing shoots from different positions of the canopy, each with more than 20 leaves and one terminal fruit cluster bearing 40–50 fruit were selected from a tree. They were randomly allocated to four treatment groups, each with five replicates consisting of five bearing shoots as the experimental plots: no girdling or defoliation (control); or girdled at a width of 5 mm at around 60 cm from the fruit cluster base and defoliated to leave 0, 5, or 10 top leaves above the girdle. The number of fruit on each panicle was counted every day until 5 days after treatment (DAT), when all fruit in the “0 leaf” group had been shed. Since girdling plus complete defoliation induced 100% fruit drop, we adopted this treatment for the other on-tree experiments in later seasons. Twenty bearing shoots with a similar sized terminal panicle were selected from different positions of five trees (*n* = 5, one tree as one experimental block) and girdled and defoliated 50 days after anthesis as mentioned above, and 20 untreated panicles with similar fruit load from each tree used as controls. Fifteen of them were used for daily sampling, and five of them used for tracing abscission through daily counting of fruit in each panicle. Fifty fruit per treatment from each tree were collected every day and dissected into fruit and pedicel for analyses of sugars and enzyme activities.

For the off-tree experiment, our initial trial showed that detached fruit desiccated quickly and never shed. However, if the peduncle of the fruit cluster was inserted into distilled water immediately after detachment from the tree to prevent desiccation, intensive fruit abscission occurred in a few days. Therefore, water-fed detached fruit clusters each with over 20 fruit were used to examine the effects of the H_2_O_2_ scavenger dimethylthiourea (DMTU) on abscission and cellulase activity in the abscission zone. The treatment used DMTU solution (0.1%, w/v) to replaced distilled water used in the control group. All the clusters were placed in an incubator (RXZ-0450, Jiangnan, Nibo, China) at 28°C, 85% relative humidity, and a 12/12 dark–light cycle with a light intensity of 40 μmol m^–2^s^–1^ during light period. Five clusters in each treatment were set for daily recording of fruit abscission, and five other clusters were set for sample taking. Pedicels were collected from four to five fruit in each cluster every day for measurement of cellulase activity in the abscission layer. The experiments had five replicates consisting of samples from the five clusters.

### Sugar Contents in the Fruit and Pedicel

Fruit and pedicel samples were taken from the on-tree experiment. Sugars were extracted from fruit or pedicel tissues of known weight with 90% (v/v) ethanol solution and analyzed using high performance liquid chromatograph (HPLC) according to Wang et al. (2006).

### Observation of ROS Occurrence Around the Abscission Zone in the Pedicel by Confocal Microscopy

The occurrence and tissue distribution of ROS was analyzed using the H_2_O_2_ fluorescent probe 2′,7′-dichlorofluorescin diacetate (DCFH-DA; Coelho et al., 2002). DCFH-DA is converted into DCFH that reacts with one-electron oxidizing species including •OH generated from Fenton reduction of H_2_O_2_ instead of with H_2_O_2_ *per se* and generates fluorescence-emitting DCF* (Kalyanaraman et al., 2012). Therefore, the method indirectly reflects the abundance of H_2_O_2_. The fluorescent dye is generally considered as an intracellular ROS probe, as it requires intracellular esterase to release the reactive DCFH (Afri et al., 2004). However, the enzyme is present apoplastically in plants (Cummins and Edwards, 2004). Hence, the dye is able to probe both intracellular and apoplastic ROS in plants.

For this analysis, we chose a separate longan tree of “Chuliang” and girdled and defoliated three different bearing shoots each day from 50 days after anthesis until day 5, when fruit samples at different DAT together with the non-treated control fruit were collected. As a result, we could observe samples at different DAT on the same day, avoiding the influence of fruit age.

**FIGURE 1:**
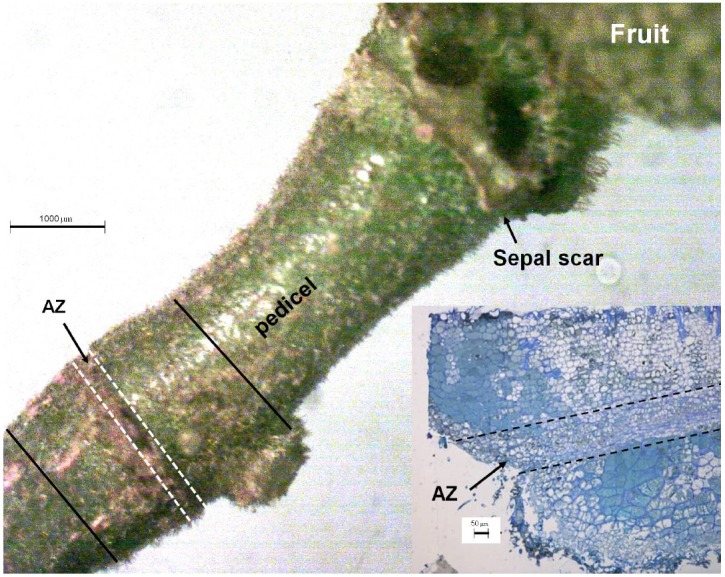
**The pedicel of a longan fruit showing the pre-existing abscission zone (AZ) with a visible sunken line (thick arrow).** The inset shows the vertical section around the AZ. The section between the solid lines was sampled for observation of tissue distribution of ROS using fluorescent dye 2′,7′-dichlorofluorescin diacetate (DCFH-DA). Abscission zone, i.e., section between the dashed lines was sampled for observation of the subcellular changes in structure and H_2_O_2_ distribution.

The abscission zone of the longan pedicel is easily identified (Figure 1). Segments of pedicel about 2 mm long including the abscission zone (between the solid lines in Figure 1) were excised, and 0.1-mm-thick vertical sections cut by hand. The sections were evacuated with a syringe in 3 mL of loading buffer (10 mmol L^–1^ Tris, 50 mmol L^–1^ KCl, and 50 μmol L^–1^ DCFH-DA, pH 7.2) with 10 μL of Triton X-100, and incubated for 30 min in the dark at 25°C. The sections were then rinsed three times with loading buffer without DCFH-DA, placed on a slide and observed under a confocal microscope (Leica TCS SP2, Mannheim, Germany), with the excitation beam at 488 nm and the emission beam at 543 nm.

### Subcellular Distribution of H_2_O_2_ in Cells of the Abscission Zone

The study was carried out with pedicels from the on-tree experiment mentioned in the section above. Pedicel disks about 0.2 mm thick comprising the abscission zone (section between dashed lines in Figure 1) were cut into slices about 0.2 mm wide and 1 mm long. The samples were prepared for observation of the subcellular distribution of H_2_O_2_ using the cerium chloride (CeCl_3_) precipitation method (Bestwick et al., 1997). The ultra-thin sections of the abscission zone were observed under a Philips FEI-TECNAI 12 transmission electron microscope (Eindhoven, Holland).

### Determination of Plasma Membrane-Bound NADPH Oxidase in the Pedicel

Purified plasma membranes were isolated from pedicel tissues by aqueous two-phase partitioning (Liu et al., 2009). Membrane protein content was estimated by the Coomassie blue G-250 protein assay using bovine serum albumin (BSA) as a standard. PM-bound NADPH oxidase was measured based on NADPH-dependent O_2_^–^ generation (Gestelen et al., 1997) using nitro-blue tetrazolium (NBT) dye, which is converted to monoformazan by O_2_^–^. This reduction was detected spectrophotometrically at 530 nm. The reaction mixture consisted of a Tris buffer (50 mmol L^–1^ Tris–HCI, pH 7.4, 250 mmol L^–1^ sucrose, 20 mM DTT, 0.1 mmol L^–1^ NBT and 0.1 mmol L^–1^ NADPH) with or without superoxide dismutase (SOD; 50 units mL^–l^). NBT reduction by O_2_^–^ was calculated from the difference in the absorbance increase rate between the presence and absence of SOD.

### Assays of Catalase (CAT), Superoxide Dismutase (SOD), and Peroxidase (POD) in the Pedicel

Pedicel tissue of known fresh weight (0.5 g) was ground into powder in liquid nitrogen added with 0.02 g PVPP. The powder was washed into a centrifuge tube with 2.5 mL ice-cold phosphate buffer solution (50 mmol L^–1^, pH 7.5) containing 0.1 mmol L^–1^ EDTA and 0.3% (v/v) Triton X-100, and centrifuged at 13,000 × *g* for 10 min at 4°C, and the supernatant was used as the crude enzyme. The protein content in the crude enzyme was determined using Coomassie blue G-250 as mentioned above.

The analysis of CAT was conducted using an oxygen electrode (Zhang and Qu, 2003). The substrate solution was a fresh 100 mmol L^–1^ H_2_O_2_ solution. Two milliliters of this substrate was transferred into the reaction well of a Hansatech Oxygraph system. When the oxygen signal in the solution had stabilized, 50 μL of crude enzyme was injected into the reaction well. The rate of oxygen release was recorded and used to calculate enzyme activity.

SOD activity was determined using a commercial assay kit provided by Najing Jiangcheng Bioengineering Institute. With xanthine–xanthine oxidase as the superoxide generator, SOD activity was quantified by the percentage of inhibition of nitroblue tetrazolium (NBT) reduction, which was recorded by optical density at 530 (OD_530_) nm. One unit enzyme activity was regarded as the inhibition of 50% of NBT reduction in 10 min. Enzyme activity per mg protein in the tissue was calculated.

POD activity was determined by guaiacol method, where 0.05 mL of the crude enzyme was added to 1 mL of 50 mmol L^–1^ phosphate buffer solution (pH = 7.0) containing 30 mmol L^–1^ H_2_O_2_ and 5 mmol L^–1^ guaiacol, and increment of absorbance at 470 nm (OD_470_) was recorded. An increment of 0.01 in OD_470_ per second was regarded as one unit of enzyme activity.

### Assay of Cellulase Activity in the Abscission Zone by Gel Diffusion

Wall-degrading cellulase in the abscission zone was analyzed by using tissue blotting and gel diffusion (Bourgault and Bewley, 2002). The substrate was sodium carboxymethylcellulose dissolved in McIlvaine buffer, pH 4.8 at 0.1% (w/v). To prepare assay gels, 100 mL of substrate was combined with 1.2 g agarose (1.2% w/v) in a 250 mL flask, boiled, cooled to approximately 65°C, and 6 mL of solution poured into a pre-warmed (65°C) set of petri dishes (60 mm in diameter). After the gel had cooled down to room temperature, 1 mm pedicel segments were cut out crosswise along the abscission line (Figure 1), and placed on the gel with the cut surface facing down. The gel with samples was incubated for 18 h at 40°C, stained for 30 min in 0.2% Congo red dye, washed for 3 min in water and then in 1 mol L^–1^ NaCl for 3 min, and fixed for 5 min in 5% acetic acid. The diameters (mm) of the transparent spots created by cellulase activity on developed gel were used to indicate relative cellulase activity.

### Statistical Analyses

Unless otherwise specified, the experiments were set out in a randomized block design using five individual trees as experimental blocks (*n* = 5). Student’s *t*-tests and least significant difference (LSD) multiple range tests (*P* < 0.05) were carried out using SPSS version 13.0 (SPSS Inc., Chicago, IL, USA).

## Results

### Effects of Girdling and Defoliation and Detachment Treatments on Fruit Abscission

**FIGURE 2:**
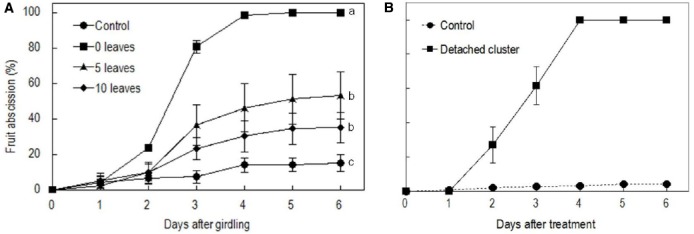
**Effect of the girdling and defoliation made on bearing shoots (A) and detachment of fruit cluster (B) on fruit abscission.** In (A), control = no girdling or defoliation; 0 leaves = girdling plus complete defoliation; 5 leaves = girdling plus defoliation leaving 5 compound leaves; 10 leaves = girdling plus defoliation leaving 10 compound leaves. Vertical bars indicate standard errors. Different letters indicate that the means are significantly different at *P* = 0.05, LSD. In **(B)**, control = on-tree fruit cluster with no girdling or defoliation treatment.

Girdling and defoliation increased fruit abscission compared with the rate in the control, and it also increased with the rate of defoliation (Figure 2A). The results suggest that the presence of source leaves determines the intensity of fruit abscission after girdling. Hence, the intensive abscission after girdling and defoliation was not a result of a wounding effect of the treatment but a result of reduced photosynthate supply due to reduced leaf number. All fruit were shed within 5 days after the treatment with girdling and complete defoliation (referring to girdling plus defoliation hereafter). The result was repeatable in the experiments in the following seasons (result not shown). Since fruit abscission in this treatment was completely predictable, we used it to study the occurrence of ROS and its related enzymes during fruit abscission. Similarly, water-fed detached clusters shed all their fruit within 5 days (Figure 2B).

### Effects of Girdling Plus Defoliation on Sugar Contents

**FIGURE 3:**
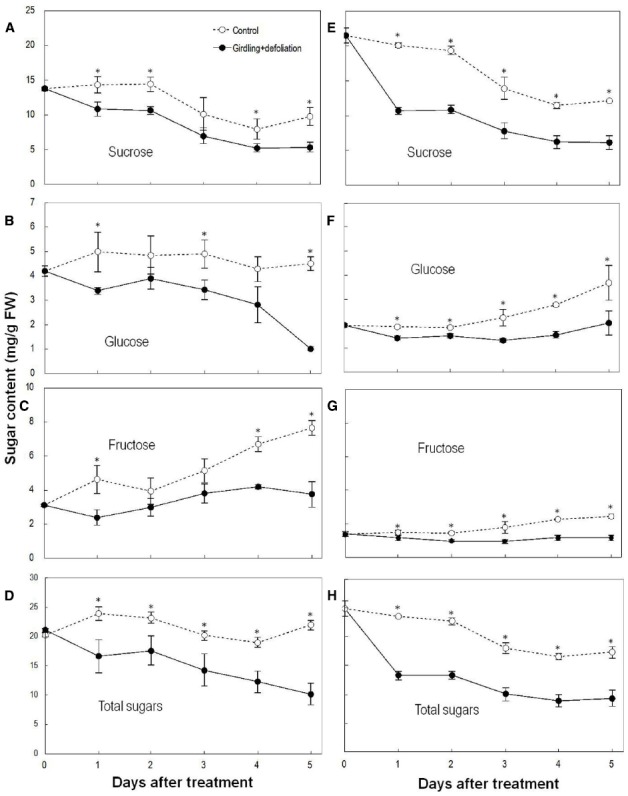
**Effect of girdling plus defoliation on the concentrations of sucrose, glucose, fructose, and total sugars in fruit (A–D) and pedicel (E–H) of longan.** Vertical bars indicate standard errors of means. Control and treatment results are indicated by dashed and solid lines, respectively. Asterisks indicate that the paired means were significantly different at *P* = 0.05, Student’s *t*-test (*n* = 5).

Sucrose was the major sugar in both the pedicel and the fruit. Girdling plus defoliation significantly decreased the contents of sugars in both parts compared with the controls (Figure 3). Sucrose showed a greater and faster decrease than glucose and fructose after the treatment. The pedicel lost nearly 50% total sugars, especially sucrose within 1 DAT, and sugar decrease was faster than in the fruit. The result confirms a carbohydrate stress is created by girdling plus defoliation.

### Occurrence of ROS in the Pedicel After Girdling Plus Defoliation Treatment

**FIGURE 4:**
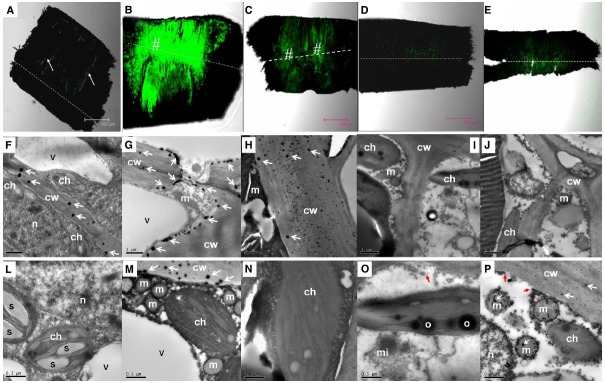
**Changes in the distribution of H_2_O_2_ as revealed by DCF fluorescence under a confocal scanning microscope in the pedicel (A–E) and by CeCl_3_ precipitation in the abscission zone under a transmission electron microscope (F–P) after girdling plus defoliation treatment. A,F,L**: samples at day 0 (control), with occurrence of H_2_O_2_ that was more concentrated in the cambium (arrow in **A**) and exclusively located in the cell walls (arrows in **F**); **B,G,M**: samples of 1 day after treatment (DAT). Note increase in fluorescence yield compared with that at Day 0 (**B** vs **A**) and apoplastic location of H_2_O_2_ along the plasma membrane (arrows) and the cell walls (arrows in **G** and **M**). **C,H,N**: samples of 2 DAT. Note fluorescence reduced compared with 1 DAT (**C** vs **B**) and H_2_O_2_ occurred exclusively in the cell walls (arrows in **H**). **D,I,O**: samples of 3 DAT, with fluorescence weaker than in the earlier samples and no observable occurrence of H_2_O_2_ in the cell walls and other cell parts. Note apparent breakdown of cytoplasm and plasma membrane (red arrow in **O**). **E,J,P**: samples of 4 DAT, with reappearance of H_2_O_2_ in the cell walls and mitochondria (white arrows in **P**) and breakdown of plasma membrane (red arrows in **P**). Dashed lines indicate the abscission layer. The symbol “#” indicates the abscission zone with relatively high H_2_O_2_. cw, cell wall; ch, chloroplast; m, mitochondrion; mi, microsome; n, nuclear; o, oil drop in chloroplast; s, starch grains in chloroplasts; v, vacuole.

In the pedicel of the control fruit, DCF fluorescence was more concentrated in the phloem and the cambium (Figure 4A), indicating constitutive production of ROS in these tissues. As revealed by CeCl_3_ precipitation, H_2_O_2_ was found exclusively in the cell walls in the cells of the abscission zone (Figure 4F). There was an abrupt increase in ROS 1 DAT, when strong DCF fluorescence was found in tissues along the abscission zone (Figure 4B). Under transmission electron microscope, H_2_O_2_ was located along the plasma membrane as well as on the cell walls in the cells of the abscission zone (Figures 4G,M). No H_2_O_2_ was observed in the chloroplasts or mitochondria (Figure 4M). The result suggests that *de novo* H_2_O_2_ production by a membrane-bound mechanism was activated by the treatment. At 2 DAT (Figure 4C), DCF fluorescence became much weaker than that on day 1. However, the abscission layer maintained high levels of fluorescence, where H_2_O_2_ was exclusively found in the cell walls (Figure 4H). Thereafter, H_2_O_2_ level decreased drastically (Figures 4D,E,I,J). Throughout the experiment, no CeCl_3_ precipitation was observed within the cytoplasm or nucleus but at 4 DAT there was some CeCl_3_ precipitation in the mitochondria as well as in the cell walls (Figures 4L–P).

In addition to changes in abundance and subcellular distribution of H_2_O_2_, some ultra-structural changes in the cells were also observed after girdling plus defoliation treatment. Starch grains observed in the chloroplasts on day 0 (Figure 4L) had disappeared by day 1 (Figure 4M). By day 3, cytoplasm breakdown had become apparent with the loss of plasma membrane integrity and disappearance of vacuole boundary (Figures 4I,J), which are signs of cell death.

### Changes in ROS Metabolism Enzymes in the Pedicel After Girdling Plus Defoliation

**FIGURE 5:**
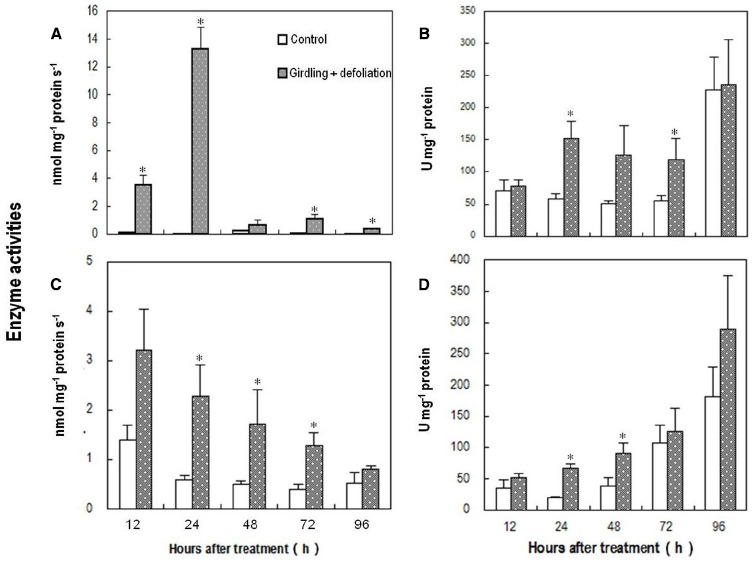
**Effect of girdling plus defoliation on the activities of PM-bound NADPH oxidase (A), superoxide dismutase (SOD) (B), catalase (CAT) (C), and peroxidase (POD) (D) in the pedicel of longan.** Vertical bars indicate standard errors. Asterisks indicate that the paired means were significantly different at *P* = 0.05, *t*-test (*n* = 5).

Coinciding with the changes in the abundance of ROS, the activity of PM-bound NADPH oxidase in the pedicel increased drastically within 12 h after girdling plus defoliation, peaked around 24 h after the treatment, and then decreased sharply although remained higher than the control (Figure 5A). The treatment also induced significant increase in SOD activity (Figure 5B), which converts superoxide anion radical generated by PM-bound NADPH oxidase into H_2_O_2._ From 24 to 72 h after the girdling plus defoliation, the activities of H_2_O_2_-scavenging enzymes, i.e., CAT (Figure 5C) and POD (Figure 5D) were significantly higher in the treated pedicel than in the control. The two enzymes displayed opposite trends, CAT decreasing while POD increasing during fruit development.

The above results show that carbohydrate stress induced an endogenous ROS burst around the abscission zone and increased the activities of both ROS generating and scavenging enzymes.

### Effects of DMTU on Fruit Abscission and Cellulase Activity in the Abscission Zone

**FIGURE 6:**
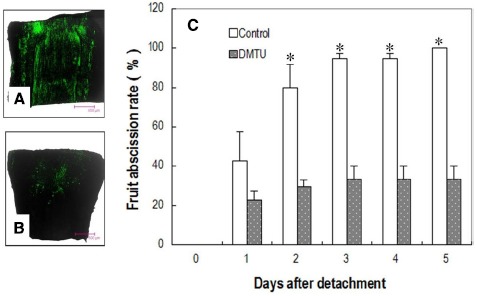
**Effect of DMTU on fruit abscission in detached longan clusters. (A)** Image of H_2_O_2_ distribution in the pedicel sampled from water-fed clusters (control) 1 day after detachment. **(B)** Image of H_2_O_2_ distribution in the pedicel sampled from dimethylthiourea (DMTU) solution-fed clusters 1 day after detachment. **(C)** Cumulative fruit abscission with days after detachment. Vertical bars indicate standard errors. Asterisks indicate that the paired means were significantly different at *P* = 0.05, *t*-test (*n* = 5).

**FIGURE 7:**
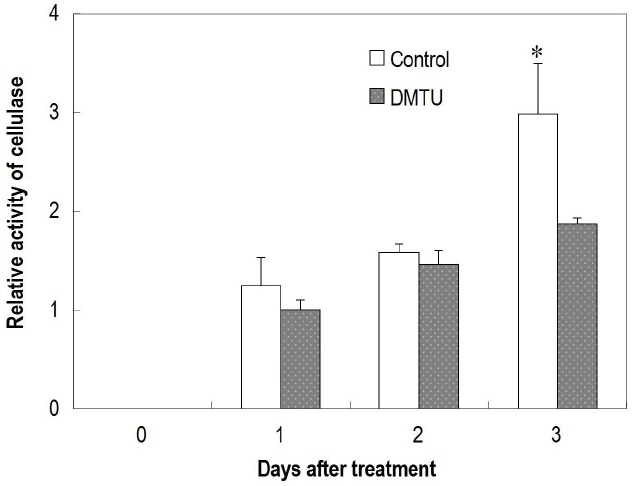
**Effect of dimethylthiourea (DMTU) on cellulase activity in the abscission zone of longan fruit in detached fruit clusters.** Vertical bars indicate standard errors. Asterisks indicate that the paired means were significantly different at *P* = 0.05, *t*-test (*n* = 5).

DMTU, which erased the H_2_O_2_ burst (Figures 6A,B), significantly suppressed fruit abscission in detached clusters (Figure 6C), suggesting H_2_O_2_ has an essential role in regulating fruit abscission. Cellulase activity in the abscission zone of the control detached fruit increased over time (Figure 7). The increase was significantly inhibited by DMTU (Figure 7), which agreed with its effect on fruit abscission.

## Discussion

Girdling is a common horticultural practice to promote flowering and fruit set, and also serves as a useful tool for physiological study of shoot behavior when it is isolated from other plant parts in terms of carbohydrate exchange (Goren et al., 2004). However, the effect of girdling on fruit set varies depending on the availability of source leaves above the girdle (Hieke et al., 2002; Figure 2A). When the supply of carbohydrates to longan fruit was cut off by girdling and complete defoliation, all the fruit abscised in a few days (Figure 2A). Similarly, detached fruit cluster deprived of carbohydrate supply shed all their fruit within 5 days (Figure 2B). Unlike the results obtained by Botton et al. (2011), who found an increase in sugar content in abscising apple fruit and suggested sugar increase as an early senescence signal in fruit that triggers abscission, girdling plus defoliation treatment induced a significant drop of all major sugars (Figure 3) and loss of starch (Figure 4) in the fruit and/or pedicel. Hence, the treatment generated a carbohydrate stress that triggered abscission. Our study was the first to explore the occurrence of ROS in response to carbohydrate stress and its role in fruit abscission.

### The Occurrence of ROS Induced by Carbohydrate Stress

Plant cells constantly produce ROS during the processes of aerobic metabolism. Several mechanisms in different cell compartments such as chloroplast, mitochondria, peroxisomes, plasma membrane, and cell walls are involved in ROS generation (Cheeseman, 2007; Sharma et al., 2012; Tripathy and Oelmüller, 2012). Results in this study showed that H_2_O_2_ occurred exclusively in the cell walls in the pedicel from non-starved fruit (day 0 sample; Figure 4F), suggesting wall-bound mechanisms were involved in normal generation of ROS. ROS is restricted to a homeostatic level in the normal cells due to the presence of ROS-scavenging mechanisms including various antioxidant molecules and enzymic processes. However, stresses such as drought, chilling, salinity, metal toxicity, UV irradiation, and pathogen attack activate the generation mechanisms and cause a burst of ROS (Lee et al., 2004; Sakamoto et al., 2008; Sharma et al., 2012). Our results provided direct evidence showing a ROS burst that occurred in response to carbohydrate stress (Figure 4). The strongest DCF fluorescence reflecting massive accumulation of one-electron oxidizing species, e.g., •OH occurred in the abscission zone at 1 DAT (Figure 4B), when H_2_O_2_ was chiefly distributed apoplastically along the plasma membrane (Figure 4G). In addition, no accumulation was found in the chloroplasts, mitochondria, or other cell parts, suggesting the outbreak of ROS caused by carbohydrate stress was generated by a membrane-bound mechanism. This coincided with a sharp increase in the activity of PM-bound NADPH oxidase (Figure 5A). Hence, this enzyme appears to be responsible for ROS generation under carbohydrate stress. A similar conclusion was obtained by Sakamoto et al. (2008) who found ROS accumulation generated by NADPH oxidase in the abscission zone of pepper leaves under salt stress. Interestingly, carbohydrate stress treatment also up-regulated ROS scavenging enzymes including SOD, catalase, and POD (Figure 5). The results clearly show that the ROS burst induced by carbohydrate stress was not a result of deactivation of scavenging mechanisms but a result of increased PM-bound NADPH oxidase.

As a mechanism that maintains ROS homeostasis, increased ROS up-regulates its scavenging enzymes (Yang and Poovaiah, 2002). The sharp decrease in PM-bound NADPH oxidase together with significant increases in H_2_O_2_-scavenging catalase and POD (Figure 5) might have led to the disappearance of H_2_O_2_ from 2 DAT. The reappearance of H_2_O_2_ on day 4 (Figure 4E) was found in the mitochondria as well as on the cell walls (Figure 4P), indicating mitochondrion-involved H_2_O_2_ generation took place in a later stage of fruit abscission.

### Roles of ROS in the Regulation of Fruit Abscission Under Carbohydrate Stress

Limited evidence is available for a role of ROS in abscission. Lai et al. (2001) found that H_2_O_2_ reduced abscission in wax apple at low temperatures. A number of other studies showed that H_2_O_2_ accumulated prior to and promoted organ abscission (Ueda et al., 1991; Sakamoto et al., 2008; Zhou et al., 2008). Sakamoto et al. (2008) found the abscission of excised pepper leaves was increased by exogenous H_2_O_2_ and decreased by H_2_O_2_ biosynthetic inhibitors or scavengers. Cohen et al. (2014) suggested that ROS was responsible for rapid root abscission in *Azolla*. In the present study, exogenous H_2_O_2_-scavenger DMTU significantly suppressed fruit abscission under carbohydrate stress. These results suggest that ROS plays an essential role in organ abscission.

The location of H_2_O_2_ in the cells displays mechanisms of its generation and provides clues of its roles in abscission. H_2_O_2_ induced by carbohydrate stress was initially found along the plasma membrane (1 DAT; Figure 4G) revealing H_2_O_2_ signal sensing at plasma membrane as well as a membrane-bound ROS generation mechanism. Later, H_2_O_2_ was exclusively located in the cell walls (Figure 4H), suggesting its actions on the cell walls. Studies thus far have indicated at least three roles for H_2_O_2_ in wall modification. First, H_2_O_2_, as a substrate, directly participates in the peroxidase-catalyzed cross-linking reactions between lignin monomers and between phenolic residues in the structural macro-molecular components of the cell walls (Brett and Waldron, 1990; Brisson et al., 1994). Second, •OH, the product of Fenton reduction of H_2_O_2_, directly breaks the polysaccharide chains, loosening the cell walls during cell elongation (Liszkay et al., 2004; Dunand et al., 2007). Involvement of •OH-mediated oxidative bond cleavage in the cell walls of the abscission zone has also been reported during rapid root abscission in *Azolla* (Cohen et al., 2014). Our observation of increased generation of DCF* from DCFH in the abscission zone after carbohydrate stress (Figure 4) is indicative of the generation of a potent •OH-like oxidant (Kalyanaraman et al., 2012). As cell separation during abscission involves both the cleavage of structural polysaccharides and lignin accumulation (Poovaiah, 1974), there is a possibility that both of the above-mentioned roles of H_2_O_2_ in wall modification are involved in the abscission induced by carbohydrate stress. Third, H_2_O_2_ was found to up-regulate the gene encoding cellulase during leaf abscission (Sakamoto et al., 2008). Our study provided further evidence of the involvement of H_2_O_2_ in the up-regulation of cellulase activity during fruit abscission (Figure 7). In addition to cell wall modifications, ROS as a plant signal may trigger a wide range of biochemical changes leading to abscission. Recently, researchers found programmed cell death (PCD) serves as a key mechanism in fruit abscission of tomato (Bar-Dror et al., 2011). There are sound evidences showing H_2_O_2_ generated by NADPH oxidase signals PCD in response to stresses (Gechev and Hille, 2005; Vannini et al., 2012; Huang et al., 2014). In the current study, accumulation of ROS in the mitochondria (Figure 4P) and signs of cell death (Figures 4O,P) in the abscission zone were observed, indicating H_2_O_2_-induced PCD in the abscission zone might be involved in fruit abscission under carbohydrate stress. Further studies are needed to clarify the signal pathways mediating ROS and abscission.

## Author Contributions

ZY and XZ conducted the major part of the experiments, data processing and writing the draft of the paper. YF took part in field data collecting, sample preparing, data processing, and draft improvement. JL, HW, and XH contributed experiment design, final data analysis and editing the manuscript. ZY and XZ contributed equally to the work.

### Conflict of Interest Statement

The authors declare that the research was conducted in the absence of any commercial or financial relationships that could be construed as a potential conflict of interest.
